# Prevalence of Leptoprosopic Type of Face among Dental Students: A Cross-Sectional study

**DOI:** 10.31729/jnma.4416

**Published:** 2019-08-31

**Authors:** Ritee Shrestha, Nitee Shrestha, Hari Prasad Upadhyay

**Affiliations:** 1 Department of Anatomy, Kantipur Dental College, Basundhara, Kathmandu, Nepal; 2 Department of Microbiology, Nepal Mediciti Hospital, Bhaisipati, Kathmandu, Nepal; 3 Department of Community Medicine, College of Medical Sciences, Bharatpur, Chitwan, Nepal

**Keywords:** *facial height*, *facial index*, *facial width*, *leptoprosopic*

## Abstract

**Introduction:**

Facial index is useful for anatomists, forensic scientists and plastic surgeons during treatment of congenital and traumatic deformities, identification of individuals in medico-legal cases and identifying craniofacial deformities and they help us in distinguishing one person from another. Very few researchers from Nepal have worked on these facial features with respect to population and environment. The study aims to find the prevalence of leptoprosopic type of face among dental students of Kantipur Dental College, Kathmandu, Nepal.

**Methods:**

The descriptive cross-sectional study was conducted among 173 dental students of age group 17-25 years in the Department of Anatomy, Kantipur Dental College Teaching Hospital and Research Center, Basundhara, Kathmandu from September to December 2018. Ethical clearance was obtained from Institutional Review Committee of the institution and convenience sampling was done. The facial parameters include facial height, facial width which was measured using Digital Vernier Caliper and Sliding Caliper respectively. The face was classified into three different types based on the value of facial index, according to Bannister Classification. Data was analyzed using SPSS 20.

**Results:**

Prevalence of leptoprosopic type of face was 70 (40.46%) [40.39-40.53 at 95%CI]. Among these, 37 (21.39%) were males and 33 (19.07%) were females. Leptoprosopic was most common which was followed by hyperleptoprosopic in 64 (36.99%), mesoprosopic in 26 (15.03%), euryprosopic in 12 (6.94%) and hypereuryprosopic facial types in 1 (0.58%). All three facial parameters including facial height, facial width and facial index were greater in male than in female.

**Conclusions:**

This study concludes that the leptoprosopic face was most common followed by hyperleptoprosopic, mesoprosopic, europrosopic and hypereuroprosopic type of face.

## INTRODUCTION

Human facial contour has always been an interesting subject for anatomists, anthropologists, plastic surgeons, and artists.^1^ Craniofacial anthropometry is used for the determination of the morphological characteristics of the head and face. Determination of facial parameters is of great importance for the evaluation of facial trauma and for easier identification of certain congenital malformations.^2^

The shape of the human face was reported to be different among different races and ethnic groups.^3^ Facial index is used in anthropometry to describe the facial proportion. Facial forms are classified according to facial index which is the percentile ratio of facial height to facial breadth.^4^ Facial index is anatomically classified as Hypereuryprosopic face (very broad face), Euryprosopic face (broad face) Mesoprosopic face (round face), Leptoprosopic face (long face) and Hyperleptoprosopic face (very long face).^5^

This study aims to determine facial index, variations in facial morphometry and face type of dental students of Kantipur Dental College and Research Center.

## METHODS

A descriptive cross-sectional study was conducted in the Department of Anatomy, Kantipur Dental College and Research Centre, Kathmandu, Nepal. After taking the ethical clearance from institutional review committee board, a data was collected from 173 students of Kantipur Dental College from September to December 2018 that includes 85 male and 88 female, of age group 17 to 25 years. Convenience (Non-probability) sampling technique was used to collect data. Sample size was calculated using the formula

Where,

n = required sample size
Z = 1.96 at 95% confidence interval
p = prevalence (27.7% from the study of Pandeya A et al^6^)
e = margin of error (10%)

$$\begin{array}{l} \text{n}=\frac{{\left(1.96\right)}^{2}}{}\frac{\times 0.277\times 0.722}{{\left(0.1\right)}^{2}}\\ =76.82 \end{array}$$

The minimum sample size required for the study was 76.82. This study was conducted among 173 students that include 85 (49.13%) males and 88 (50.87%) females. Prior to this, verbal consent was obtained from each students and the purpose of the research was conveyed to them. Further subgroup analysis was also done among two different age groups comprising of 79 (45.66%) students less than 20 years and 94 (54.34%) students greater than and equals to 20 years. The mean value of two facial parameters including facial width (FW), facial height (FH) were calculated using Spreading Caliper and VernierCaliper respectivelywith accuracy of 0.01mm and facial index (FI) was calculated as a percentile ratio of facial height by facial width. The units for the measurement of both parameters were in millimeter (mm), as mentioned in the instrument. The subject comprised of individuals with normal craniofacial configuration and without any past surgery of head and neck. Subjects with trauma of the craniofacial structures and congenital abnormalities were excluded. Subjects were told to sit upright in a relaxed mood with head in an anatomical position and without any facial expression, so that it does not alter the shape and size of face. The measurements were taken in millimetres. Following three relevant facial surface landmarks were noted:

1. Nasion: the point in the nose crossed by the midsagittal plane and naso-frontal sutures
2. Gnathion: the lowest point of mandible where the midsagittal plane intersects the lower margin of lower jaw
3. Zygion: the lateral most point on the zygomatic arch

The measurements were taken that includes:

1. Facial height (FH): distance from nasion to the gnathion
2. Facial width (FW): distance between the right and left zygion

And the value of Facial index (FI) was determined based on the formula,


Facial index(Fl): FH/FW×100

Then, the face type was classified on the basis of facial index into five different face shapes by using the Banister's classification (Table 1).^7^

**Table 1:** Banister's classification

Face type	Range of Facial Index
1. Hypereuroprosopic (very broad face)	<79
2. Europrosopic (broad face)	80-84.9
3. Mesoprosopic (round face)	85-89.5
4. Leptoprosopic (long face)	90-94.5
5. Hyperleptoprosopic (very long face)	95-99.5

The data was entered and analyzed with the help of SPSS 20 software. The descriptive data analysis was done to derive mean and standard deviation of facial width, facial height and facial index within the study participants.

## RESULTS

Prevalence of leptoprosopic type of face was 70 (40.46%) [40.39-40.53 at 95%CI]. Among these, 37 (21.39%) were males and 33 (19.07%) were females. Leptoprosopic was most common which was followed by hyperleptoprosopic in 64 (36.99%), mesoprosopic in 26 (15.03%), euryprosopic in 12 (6.94%) and hypereuryprosopic facial types in 1 (0.58%) (Table 2).

**Table 2:** Distribution of face type among study population.

Face types	FI	Male n (%)	Female n (%)	Total n (%)
Hypereuroprosopic	<80	0 (0)	1 (0.58)	1 (0.58)
Europrosopic	80-85	1 (0.58)	11 (6.36)	12 (6.94)
Mesoprosopic	85-90	13 (7.51)	13 (7.51)	26 (15.03)
Leptoprosopic	90-95	37 (21.39)	33 (19.07)	70 (40.46)
Hyperleptoprosopic	>95	34 (19.65)	30 (17.35)	64 (36.99)
Total		85 (49.13)	88 (50.87)	173 (100)

The present study was conducted among 173 dental students, of which 85 (49.13%) were males and 88 (50.87%) were females. It was also done among two different age groups comprising of 79 (45.66%) students less than 20 years and 94 (54.34%) students greater than and equals to 20 years. The mean value of FH, FW and FI were found to be 111.73±6.34 mm, 119.97±7.92 mm and 93.36±5.79 respectively in total population (Table 3).

**Table 3:** Overall comparison of all three facial parameters (n= 140).

Facial Parameters	Mean	S.D.			95% CI of the difference	
			**Minimum**	**Maximum**	**Lower**	**Upper**
FH	111.73	6.34	97.35	127.65	110.77	112.68
FW	119.97	7.92	103.32	146.69	118.77	121.15
FI	93.36	5.79	81.25	112.43	92.49	94.23

The mean of all three parameters were determined in two age groups and genders shown (Table 4 and Table 5). All the parameters were found to be greater in students above twenty years and in male students, which suggest that all three parameters increases after the age of twenty and there occurs a sexual dimorphism in all three parameters. This study revealed higher values FH, FW and FI in males compared to females. Similarly, the leptoprosopic type of face was found to be most common in both male and female including both the age groups.

**Table 4:** Distribution of face type among two different age groups.

Age group	Number of students	FH	FW	FI	Face types
**<20**	79	110.86±6.57	119.51±7.77	92.96±5.67	Leptoprosopic
**≥20**	94	112.77±5.92	120.50±8.10	93.84±5.93	Leptoprosopic

**Table 5:** Distribution of face type among two different genders.

Gender	Number of students	FH	FW	FI	Face types
**Male**	85	112.58±6.40	120.52±7.63	93.54±4.29	Leptoprosopic
**Female**	88	110.91±6.20	119.43±8.19	93.19±6.97	Leptoprosopic

## DISCUSSION

A study conducted among 173 dental students showed that, 85 (49.13%) were males and 88 (50.87%) were females. Present study revealed that mean and SD of FH, FW and FI in dental students were 111.73±6.34 mm, 119.97±7.92 mm and 93.36±5.79 mm respectively. In general, females showed the minimal measurement as compared to males in all three facial parameters. The mean and SD of FH and FW were 112.58±6.4 mm and 120.52±7.63 mm respectively in male and 110.91±6.20 mm and 119.43±8.19 mm respectively in female. In a study done by Yesmin T in Malay population, the mean and SD of FH and FW were found to be 117.7±7.99 mm and 129.9±7.7 mm respectively in male and 107.00±8.4 mm and 125.0±7.5 mm respectively in females. It suggests that both FW and FH is found to be higher in malay males whereas only FW is higher in malay males compared to Nepalese people.

In a study done by Omotoso DR in Bini tribe of

Nigeria, in four different age groups 16-20 years, 2125 years, 26-30 years and 31-35 years FI in male and female were 87.98±2.55 mm and 85.88.8±2.48 mm respectively.^9^ It suggests that FI is greater in both male and female in Nepali population than in Nigeria. In a study carried by Datta S and Sawant VG in a people of Maharastra, India, FH, FW in male were 103.4±9.2mm and 120.9±6.5mm respectively and in female is 111.9±8.7 mm and 129.0±7.4 mm,^10^ which suggests that both FH and FW are higher in maharastrian female and but only FH is greater in maharastrian male compared to Nepalese population.

Facial Index of Gujarati population of age group 18 to 25 years was found to be 81.7±4.2 mm and most common facial type is europrosopic being 42.96% followed by hypereuroprosopic being 35.1% and none of them have hyperleptoprosopic type.^10^ In contrast to our study in which the least facial type is hypereuroprosopic being 0.58% followed by europrosopic being 6.94% and hyperleptoprosopic is second most common facial type being 36.99%.

Similarly, in a study done by Pandeya A and Atreya A in students of Lumbini Medical College, the FH and FW were 113.1±4.7 mm and 126.9±6.4 mm respectively in male and 104.1±6.8 mm and 121.4±6.0 mm respectively in female. The mean values of FH, FW and FI in total population were 108.3±7.4 mm, 123.9±6.7 mm and 87.44±4.82 respectively. All three parameters were found to be more than our findings in total population. Most common type of face was Mesoprosopic, ^6^ whereas the most common type of face in our study is leptoprosopic, which was the second most common type of face in their study.

It has been shown that males in the studied population of the central part of Serbia done by Jeremic D et al that male have significantly higher values of FH, FW and FI, compared to the females. The mean values of the FH, FW and FI in males were respectively, 121.42±5.79 mm, 129.12±8.86 mm, 94.04±7.00 mm, while the following values were obtained in females as 110.84±5.61 mm, 119.98±6.38 mm, 92.38±6.72 mm respectively, whose values in male were higher than our study and were almost equal to our findings in female. The dominant type of face in the studied population was leptoprosopic with an incidence of 81.71%, which was similar to our findings with an incidence of 40.46%. In the Serbian population hypereuroprosopic and europrosopic were not observed in both genders, but in our study hypereuroprosopic was observed only in 0.58% and europrosopic in 0.58% and 6.36%.

In a study conducted by Ahim AA et.al, in Kudrish population of Sulaimani city showed that the mean values of FH, FW and FI were 109.4±8.35 mm, 120.01±9.06 mm and 91.05±9.54 mm respectively in males and 101.5±8.35mm, 112.01±9.06mm, 90.05±9.7 mm in females, which revealed higher values of FH, FW and FI in males compared to females. According to the value of FI, the dominant type of face phenotype was leptoprosopic with a prevalence of 50.5% which corresponds to our finding as leptoprosopic with a prevalence of 40.46%.^11^ Standard comparison parameters of facial index between North Indian males (101.04±0.95) and South Indian males (100.28±1.77) and between North Indian females (107.70±7.69) and South Indian females (85.39±6.33), done by Prasanna LC and Mamatha H, all four values of FI were found to be greater in Indian populations than in Neplaese.^12^

In a study done on Purana population by Chandimal KM, the mean FH were 125.6±9.3 mm and 120.0±6.4 mm in male and female respectively and FW were 120.0±6.4 mm and 109.9±7.7 mm in male and female, whose values in both genders were higher than the values obtained in our study. Similarly, mean FI of male and female was (92.01±5.10) and (89.99±6.20) respectively, whose values in both genders were less than our findings. The mean FI of male was higher than the female. The mean FI of total population was 90.6±6.41 irrespective of the gender and Facial phenotyping according to the FI showed that the large majority of the subjects belong to leptoprosopic (37.66%) category followed by hyperleptoprosopic (23.05%), mesoprosopic (22.72%), europrosopic (9.74%) and hypereuroprosopic (6.8%),^13^ whose facial type prevalence was found to similar to our finding in which highest being to leptoprosopic (40.46%) followed by hyperleptoprosopic (36.99%), mesoprosopic (15.03%), europrosopic (6.94%) and ypereuroprosopic (0.58%).

The limitation of our study includes the less number of subjects with unequal distribution of population in two different age groups and genders. The sampling was done by convenient sampling technique so the result cannot be generalised. Although all the measurements were done meticulously there might be slight difference measurements obtained by other person. To increase the validity of result, random sampling must be done with larger population setting.

## CONCLUSIONS

This study concludes that leptoprosopic face was most common followed by hyperleptoprosopic, mesoprosopic, europrosopic and hypereuroprosopic type of face.
